# Supplementary figures and images for: Molecular and microscopic detection of natural and experimental infections of Toxocara vitulorum in bovine milk
Source: PLoS One. 2020 May 20;15(5):e0233453. doi: 10.1371/journal.pone.0233453 (PMC7239449; doi:10.1371/journal.pone.0233453)

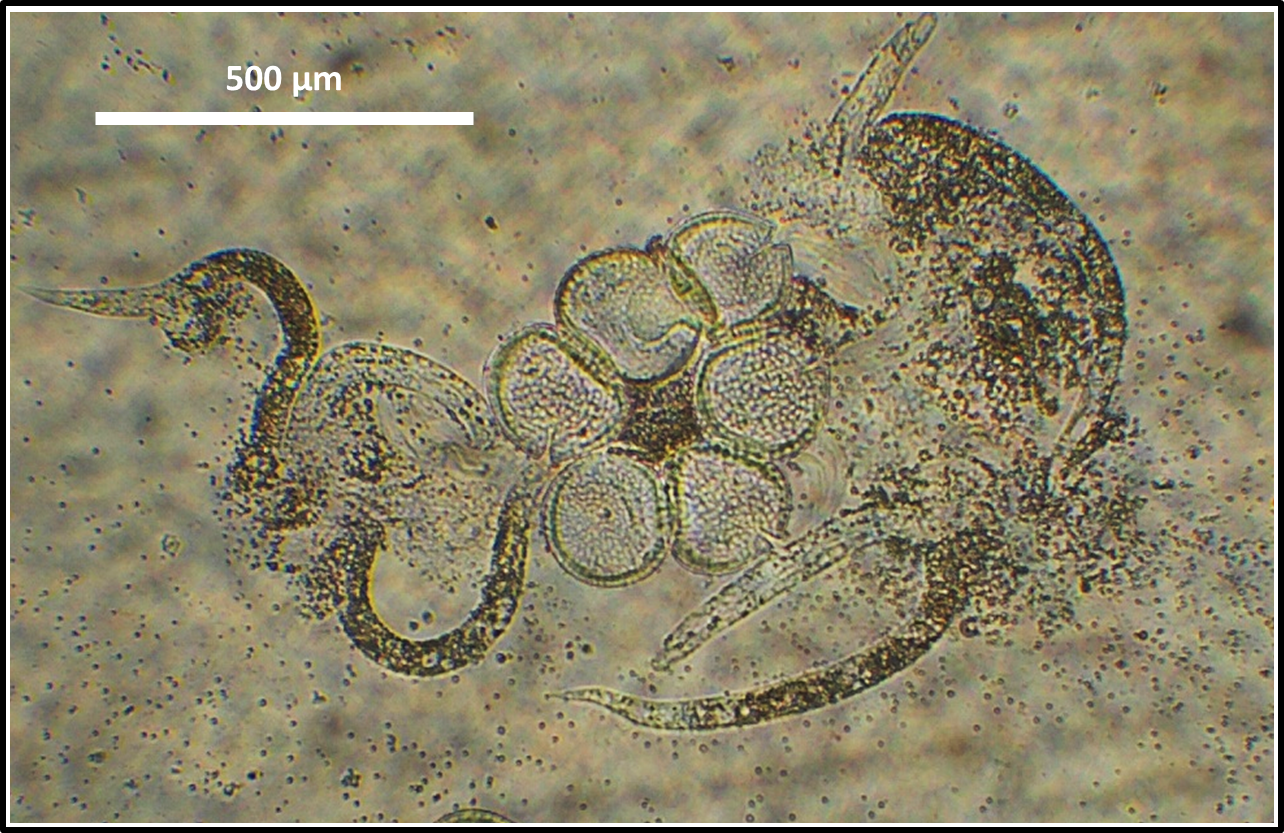

Supplement: S1 Fig — (TIF) [file pone.0233453.s002.tif]

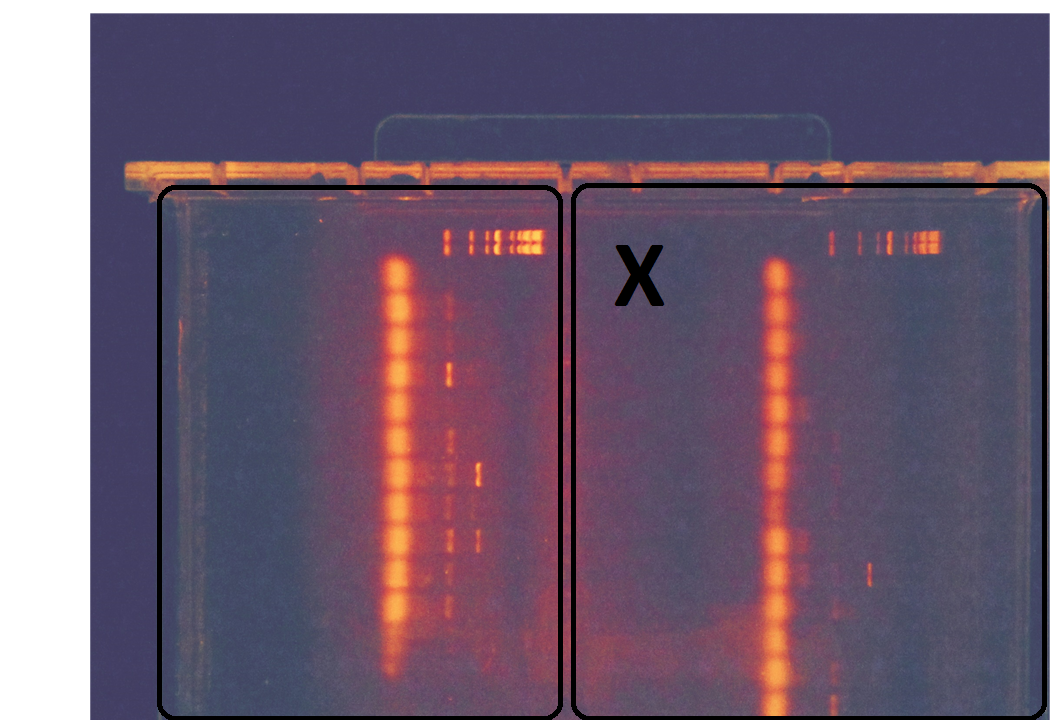

Supplement: S2 Fig — (TIF) [file pone.0233453.s003.tif]
