# Supplementary material for: Molecular and microscopic detection of natural and experimental infections of Toxocara vitulorum in bovine milk
Source: PLoS One. 2020 May 20;15(5):e0233453. doi: 10.1371/journal.pone.0233453 (PMC7239449; doi:10.1371/journal.pone.0233453)
Supplement: Attachment — Submitted filename: Review_PONE-D-19-34351.docx [file pone.0233453.s004.docx]

Ref: PONE-D-19-34351
Title: Molecular and microscopic detection of natural and experimental infections of *Toxocara* vitulorum in bovine milk

The objective of this study was to detect the experimental and natural infection with *Toxocara vitulorum* larvae in the bovine milk applying two techniques, the classical microscopy-based centrifuge-sedimentation method and the more advanced molecular-based PCR assay.

The manuscript is well written, and the results are very interesting.

The paper is accepted after **minor revision**.

However, see comments below:

**Abstract**

Line 11: replace “*T. vitulorum”* with “*Toxocara vitulorum”*

**Introduction**

Line 41: Please, put *T. vitulorum* between two brackets and remove the commas.

**Material and Methods**

Lines 96-106: Insert the bibliographic reference of the method used.

Lines 106-119: Insert the bibliographic reference of the method used.

Lines 119-129: Insert the bibliographic reference of the method used.

Line 151: Explain if the 50 animals have undergone copromicroscopic examination to know their positivity to *T. vitulorum*.

Lines 232-234: add for each stage the time (days) of development of the larvae in the egg.

Line 234: In the legend of Fig. 2, remove "Scale bar is 50 μm" and add "50 μm" on the white bar in the photos.

**Results**

Line 284: Add a photo of larvae identified with the optical microscope.

**Discussion**

Lines 297-298: The bibliographic references cited are very outdated, use or add more updated ones.

Line 358: cursive writing *in vitro.*

**General comments**:

The overall paper is well written, but I think you should add more bibliographic references regarding the methods used in laboratory tests.

Moreover, bibliographic references of the introduction and discussion should be more up to date.
