# Supplementary material for: Molecular and microscopic detection of natural and experimental infections of Toxocara vitulorum in bovine milk
Source: PLoS One. 2020 May 20;15(5):e0233453. doi: 10.1371/journal.pone.0233453 (PMC7239449; doi:10.1371/journal.pone.0233453)
Supplement: Attachment — Submitted filename: Response to Reviewers.docx [file pone.0233453.s005.docx]

**April 7^th^, 2020**

Jacopo Guccione
Academic Editor
PLOS ONE

**PONE-D-19-34351
Molecular and microscopic detection of natural and experimental infections of Toxocara vitulorum in bovine milk**

Dear Dr. Guccione

Thank you for the reviews by you and the two reviewers of our manuscript “Molecular and microscopic detection of natural and experimental infections of *Toxocara vitulorum* in bovine milk”. We have since revised the manuscript according to the reviewer’s comments, which are detailed in the following for your perusal:

**Reviewer #1:**

The manuscript PONE-D-19-34351 reports the results of a study aimed at detecting the experimental and natural infection with Toxocara vitulorum larvae in the bovine milk applying two techniques, i.e. the classical microscopy-based centrifuge-sedimentation method and a molecular-based PCR assay.

The experimental set-up permitted to recover larvae of T. vitulorum from milk samples both after experimental contamination and from colostrum samples of naturally infected animals.

I recommend the publication of the paper after major revisions. The questions posed by the authors are well defined, the methods appropriate and the results, discussion and conclusions well balanced and adequately supported by the data. However, there some issue which should be addressed by the Authors.

- Introduction is confusing and with too many paragraphs on Toxocara canis and T. cati (e.g. lines 62 to 72)

**Response:** This is due to the matter of fact that the dog and cat Toxocara species are the most studied, and to pin down the fact that T. vitulorum did received the least attention despite its economic and medical importance. Thus it highlights the importance of our study, and why it was initiated.

- In the introduction, I would suggest to provide prevalence of T. vitulorum in different geographical areas

**Response:** A paragraph describes the prevalence of T. vitulorum in different geographical areas has since been added to the “Introduction” section (Lines: 48-55).

- Figure 1 is not necessary to the MS. I would suggest removing it.

**Response:** Figure 1 has since been removed, as per our agreement with the reviewer’s recommendation.

- Line 190. I think that “Mancianti et al., 2013” is not an appropriate reference for Kappa coefficient ranking. I suggest the authors to use a statistical reference.

**Response:** A more specialized statistical reference has been applied to cite the statistical method of “Kappa coefficient”. I would thank the reviewer for pinpointing the correction.

- In the discussion the authors mention microscopy and molecular methods for detection of larvae in milk samples, as well as antibody-based serological methods. What about antigen-based milk ELISA methods? And what about copromicroscopic methods?

**Response:** Unfortunately and after deep and rigorous mining of all publications on T. vitulorum, only antibody-based serological methods had been used to monitor excretion of parasite in the colstrum/milk samples, with no data on the antigen-based milk ELISA. Also since our study was only focused on the milk excretion with no interest of faecal excretion data, the copromicroscopic methods become of less significance to be mentioned in the “Discussion” part. But again I would appreciate the reviewer for raising the point here.

• Furthermore, do the author have any clue regarding the costs of each diagnostic method?

**Response:** In short, the PCR-based molecular method would be more expensive than the microscopic examination after the concentration-sedimentation technique (would approximate 3000 Egyptian pound/180 USD per 50 samples versus ~ 800 EGP/50 USD, respectively). Albeit, the molecular method still to give more sensitive detection levels when compared with the microscopy method when applied in the experimental part of the study. Thus, and again as mentioned in the “Discussion” part, the molecular technique would fit more the research purposes, while the microscopic examination would constitute a golden standard for surveying the milking animals.

I shall thank the reviewer for recommending these constructive corrections, which with no doubt have added much to the manuscript, leading to much improvement in its quality and readability.

**Reviewer #2**

Title: Molecular and microscopic detection of natural and experimental infections of Toxocara vitulorum in bovine milk.
The objective of this study was to detect the experimental and natural infection with Toxocara vitulorum larvae in the bovine milk applying two techniques, the classical microscopy-based centrifuge-sedimentation method and the more advanced molecular-based PCR assay.
The manuscript is well written, and the results are very interesting.
The paper is accepted after minor revision.
However, see comments below:

- **Abstract:**
- Line 15: replace “T. vitulorum” with “Toxocara vitulorum”

**Response:** It has been corrected

- **Introduction:**
- Line 43: Please, put T. vitulorum between two brackets and remove the commas.

**Response:** It has been corrected

- **Material and Methods**
- Lines 96-106: Insert the bibliographic reference of the method used.

**Response:** A suitable bibliographic reference of the method used has been inserted.

- Lines 106-119: Insert the bibliographic reference of the method used.

**Response:** The method used was based solely on our trials and observations during the pilot and preliminary experiments in the Lab. It has been written in enough details so giving the fellow researchers the opportunity of copying it into their Labs.

- Lines 119-129: Insert the bibliographic reference of the method used.

**Response:** The method used was based solely on our trials and observations during the pilot and preliminary experiments in the Lab. It has been written in enough details so giving the fellow researchers the opportunity of copying it into their Labs.

- Line 151: Explain if the 50 animals have undergone copromicroscopic examination to know their positivity to T. vitulorum.

**Response:** No, Milk/Colostrum samples were collected blindly from 50 animals, without performing any copromicroscopic examination. This is because the main specific aim was to compare the same two methods blindly on the same milk samples, as it is not necessarily that the same copro-positive animal to excrete the parasite in its milk.

- Lines 232-234: add for each stage the time (days) of development of the larvae in the egg.

**Response:** Information about developmental periods (in days) has been added.

- Line 234: In the legend of Fig. 2, remove "Scale bar is 50 μm" and add "50 μm" on the white bar in the photos.

**Response:** It has been corrected on the photos.

- **Results**
- Line 284: Add a photo of larvae identified with the optical microscope.

**Response:** Supporting files (Supporting video and figure) have been added to show the viable larvae inside the fully-embryonated eggs, as well as larvae being extracted from mechanically-broken eggs.

- **Discussion**
- Lines 297-298: The bibliographic references cited are very outdated, use or add more updated ones.

**Response:** More updated references have been added accordingly, many thanks for the reviewer for his recommendation.

- Line 358: cursive writing in vitro.

**Response:** It has since been corrected.

- **General comments:**
  The overall paper is well written, but I think you should add more bibliographic references regarding the methods used in laboratory tests.
  Moreover, bibliographic references of the introduction and discussion should be more up to date.

**Response:** As it can be easily figured out that the parasite of T. vitulorum is considered as a neglected when compared to the heavily-researched counterparts of dog and cat ascarids, and thus just barely over 20 papers had been published throughout the last decade. Nevertheless, more updated references have been added to the introduction and discussion sections, to reflect upon the reviewer’s recommendations.

We finally would greatly appreciate the reviewer for his comments which led to much improvement in the quality of the manuscript.

In all, we consider the paper much improved after the suggested revisions, and would like to thank you and the reviewers for your kind help. We do hope that the paper is now deemed acceptable for publication in PLoS One.

With my best regards,

Sincerely,

Mohamed Bessat, Ph.D.

Associate Professor of Parasitology

Faculty of Veterinary Medicine, Alexandria University

Egypt
